# *Colletotrichum truncatum* Causing Anthracnose of Tomato (*Solanum lycopersicum* L.) in Malaysia

**DOI:** 10.3390/microorganisms11010226

**Published:** 2023-01-16

**Authors:** Saleh Ahmed Shahriar, Asmaul Husna, Terna Tersoo Paul, Most. Nurjahan Khatun Eaty, Md Quamruzzaman, Abu Bakar Siddique, Md Abdur Rahim, Abu Noman Faruq Ahmmed, Jasim Uddain, Shafiquzzaman Siddiquee

**Affiliations:** 1 School of Biological Sciences, Universiti Sains Malaysia, Gelugor 11800, Penang, Malaysia; 2 Department of Plant Science and Biotechnology, Federal University of Lafia, PMB 146, Lafia 950101, Nasarawa State, Nigeria; 3 College of Agricultural Sciences, IUBAT–International University of Business Agriculture and Technology, Dhaka 1230, Bangladesh; 4 Tasmanian Institute of Agriculture, University of Tasmania, Prospect 7250, Australia; 5 Department of Genetics and Plant Breeding, Sher-e-Bangla Agricultural University, Dhaka 1207, Bangladesh; 6 Department of Plant Pathology, Sher-e-Bangla Agricultural University, Dhaka 1207, Bangladesh; 7 Department of Horticulture, Sher-e-Bangla Agricultural University, Dhaka 1207, Bangladesh; 8 Biotechnology Research Institute, Universiti Malaysia Sabah, Kota Kinabalu 88400, Sabah, Malaysia

**Keywords:** anthracnose, *Colletotrichum truncatum*, *Solanum lycopersicum*, morphology, molecular identification, pathogenicity

## Abstract

Tomato (*Solanum lycopersicum* L.) is a popular nutritious vegetable crop grown in Malaysia and other parts of the world. However, fungal diseases such as anthracnose pose significant threats to tomato production by reducing the fruit quality and food value of tomato, resulting in lower market prices of the crop globally. In the present study, the etiology of tomato anthracnose was investigated in commercial tomato farms in Sabah, Malaysia. A total of 22 fungal isolates were obtained from anthracnosed tomato fruits and identified as *Colletotrichum* species, using morphological characteristics. The phylogenetic relationships of multiple gene sequence alignments such as internal transcribed spacer (ITS), β-tubulin (*tub2*), glyceraldehyde 3-phosphate dehydrogenase (*gapdh*), actin (*act*), and calmodulin (*cal*), were adopted to accurately identify the *Colletotrichum* species as *C. truncatum*. The results of pathogenicity tests revealed that all *C. truncatum* isolates caused anthracnose disease symptoms on inoculated tomato fruits. To our knowledge, the present study is the first report of tomato anthracnose caused by *C. truncatum* in Malaysia. The findings of this study will be helpful in disease monitoring, and the development of strategies for effective control of anthracnose on tomato fruits.

## 1. Introduction

Tomato (*Solanum lycopersicum* L.) belongs to the Solanaceae family, and is popular for its huge nutritious and economic value. A variety of diseases attack tomato fruits and plants, including major fungal diseases that threaten tomato production globally, such as anthracnose, early blight, late blight, leaf mold, septoria leaf spot, powdery mildew, fusarium wilt, and verticilium wilt [1]. *Colletotrichum* spp. are important plant pathogens, causing anthracnose diseases in a diverse range of host plants, including vegetables, fruits, legumes, cereals, herbaceous, conifers, woody, and ornamental plants, at both developing and mature stages of plant growth [2,3,4]. Some taxa are restricted to certain host species, or cultivars, while others have extensive host ranges [2,4,5]. *Colletotrichum* spp. are commonly associated with tomato anthracnose of which *C. truncatum* has been reported as an emerging pathogen causing huge yield losses of tomato annually.

Differentiation of *Colletotrichum* spp. on the basis of host associations alone is not a reliable criterion for species identification, because a few taxa such as *C. acutatum*, *C. dematium*, and *C. gloeosporioides*, infect a wide range of plant hosts. Therefore, taxonomic classification of *Colletotrichum* species has primarily focused on identification and characterization of sub-populations within the species [6,7,8]. The conventional identification and characterisation of *Colletotrichum* species mainly relied on morphological differences of wide variety of isolates from ample ranges of host crops. However, morphological characteristics alone are also not reliable for identification of *Colletotrichum* species, due to a variety of variables such as the environment, which influences the stability of the morphological traits and the coexistence of intermediate forms in nature [9].

PCR tests and DNA sequence alignments from multiple genes have been widely utilized to overcome the limitations of morphological characterisation in accurate species delineation [10], and data generated from nucleic acid tests have provided a reliable framework for building the taxonomic classification of *Colletotrichum* species [11]. A study by Photita et al. [12], showed that sequence analysis based on ITS regions are helpful in determining the phylogenetic relationships within the *Colletotrichum* species [12]. Apart from the ITS region, partial *tub2*, *gapdh*, *act* and *cal* genes sequence analyses have also been employed to resolve the phylogenetic relationships within the *C. truncatum* species [13,14]. The utilisation of morphological studies coupled with sophisticated molecular data has proven to be an efficient method in identifying *C. truncatum* isolates and has increased the understanding of its taxonomy [2,9]. Thus, in the present study, polyphasic identification involving morphological and molecular characterisation was adopted for the substantive identification of *C. truncatum* isolates recovered from diseased tomato fruits. Pathogenicity tests were also conducted to assess the pathogenic ability of the *C. truncatum* isolates on artificially inoculation tomato fruits.

## 2. Materials and Methods

### 2.1. Sample Collection and Fungal Isolation

Tomato fruit samples showing typical anthracnose symptoms were collected from three commercial tomato gardens in Sabah, Malaysia. The samples were placed in zip-lock plastic bags, and conveyed to the Biotechnology laboratory of Universiti Malaysia Sabah for fungal isolation. Diseased tissues were cut into smaller pieces of about 1 cm^2^, and surface-sterilised by soaking in 70% ethanol for 3 min, followed by 1% sodium hypochlorite for 3 min, and rinsed for 1 min each in three changes of sterile distilled water. The sterilised samples were then placed on sterile potato dextrose agar (PDA) medium and incubated under room temperature (25 ± 2 °C) for one week, to obtain fungal mycelial growths. The resulting fungal mycelia were sub-cultured on new PDA plates, and pure cultures of fungal isolates were obtained following the single conidium isolation method previously reported by Zhang et al. [15].

### 2.2. Morphological Characteristics

Fungal isolates obtained were cultured onto PDA plates and incubated at 25 ± 2 °C for 7 days. The macroscopic characteristics such as colony appearance; pigmentation; and mycelial growth rate were recorded. For microscopic characteristics, the arrangement, shape, and size of acervuli; conidia; conidiogenous cells; appressoria; and setae were examined. Preliminary identification was in accordance with the fungal descriptions of Cabrera et al. [16].

### 2.3. Extraction of Genomic DNA, PCR Amplification, and DNA Sequencing

All isolates were cultured on potato dextrose broth (PDB) and incubated at 25 ± 2 °C for 5 days. After incubation, the fungal mycelia were harvested from the broth cultures, dried on sterile filter papers, and homogenized into fine powder, using liquid nitrogen. A total of 60 mg of the fine powder was transferred into a 1.5 mL microcentrifuge tube for DNA extraction using Invisorb Spin Plant Mini Kit (Stratec, Birkenfeld, Germany), following the manufacturer’s instructions. DNA samples were preserved at –20 °C for PCR amplifications. The extracted genomic DNA samples were subjected to PCR amplifications in Thermal Cycler (Biorad, Hercules, CA, USA) using five primer pairs, ITS (ITS1/ITS4), *tub2* (Bt2a/Bt2b), *gapdh* (GDF1/GDR1), *act* (ACT-512F/ACT-783R) and *cal* (CAL-228F/CAL-737R) (The primer sequences are provided in Table 1). The amplification reactions were carried out in a total volume of 50 μL consisting 8 μL Green GoTaq^®^ Flexi Buffer (Promega, Madison, WI, USA), 8 μL MgCl2 solution (Promega, Wisconsin, USA), 1 μL dNTP mix (Promega, Wisconsin, USA), 8 μL of each primer (Promega, USA), 0.3 μL GoTaq® DNA polymerase (Promega, Wisconsin, USA), 1 μL genomic DNA, and sterile distilled water to make up a total volume of 50 μL.

PCR reactions were carried out in a MyCyclerTM Thermal Cycler (Bio-rad, Hercules, CA, USA), with initial denaturation at 95 °C for 5 min, followed by 32 cycles of denaturation at 95 °C for 30 s, annealing at 56 °C for 30 s, and extension at 72 °C for 1 min. Final extension was performed at 72 °C for 10 min. The PCR products were detected in agarose gel electrophoresis (1%), and sent to a service provider (First BASE Laboratories Sdn Bhd, Seri Kembangan, Malaysia) for DNA purification and sequencing.

### 2.4. Sequences Alignment, BLAST, and Phylogenetic Analysis

The forward and reverse DNA sequences obtained were aligned using the Molecular Evolutionary Genetics Analysis (MEGA) software, version 11, to create a consensus sequence for each isolate [22]. The identity of the fungal isolates was determined based on the highest percentage of sequence similarity on GenBank, using the Basic Local Alignment Search Tool (BLAST). Multiple sequence alignments of ITS region, *tub2*, *gapdh*, *act* and *cal* genes were performed to determine the fungal species and their phylogenetic relationships. The phylogenetic tree was constructed using the maximum likelihood (ML) method on the MEGA11 software. For the ML method, a model test was run to select the best nucleotide substitution model. Kimura 2-parameter + gamma distribution (K2 + G) model was adopted to construct a robust phylogenetic tree, and the robustness of the tree was evaluated using a bootstrap analysis with 1000 replicates.

### 2.5. Pathogenicity Assays

The pathogenicity of all obtained fungal isolates was assessed on healthy fruits of tomato using the wound inoculation method previously described by Cabrera et al. [16].

Fungal isolates were cultured on PDA for 7 days at 25 ± 2 °C, and fungal conidial suspensions were prepared by flooding the culture plates with sterile distilled water. A sterilized glass spreader was used to extricate conidia, and the concentration was adjusted to 1 × 10^6^ conidia/mL using a haemocytometer (Weber, Teddington, UK). Prior to inoculation of wounded fruits, disease-free fruits of tomato were surface-sterilized by swabbing with 70% ethanol, the surface-sterile fruits were wounded by pricking with a sterile toothpick, and inoculated by applying sterile cotton wools immersed in the prepared conidial suspensions (~200 μL) at the wounded sites. Wounded fruits inoculated with sterile distilled water served as control.

All inoculated fruits were placed in a plastic tray and sealed with a transparent plastic wrap. The trays were kept humid by placing petridishes containing water inside the tray to maintain approximately 80% relative humidity. Symptoms that developed on inoculated fruits were observed and recorded. After 7 days of inoculation, the lesion area was measured and recorded. Differences in the lesion area were determined by one-way analysis of variance, and means were compared by the Tukey’s test at 5% level of probability, using the IBM SPSS Statistics software version 26. Fungal isolates were re-isolated from the symptomatic inoculated fruits of tomato and re-identified based on the morphological characteristics of the original cultures to confirm Koch’s postulates.

## 3. Results

### 3.1. Disease Survey

**Figure 1:**
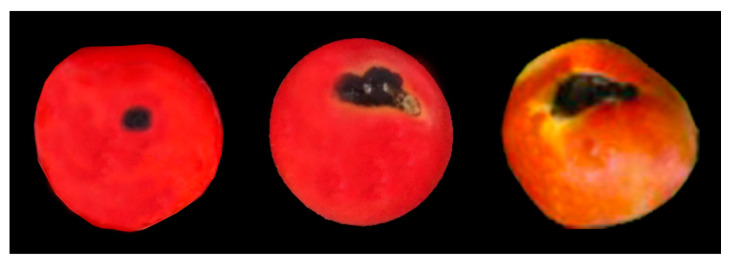
Symptoms of tomato anthracnose observed in the tomato gardens in Sabah, Malaysia.

Typical symptoms of anthracnose disease were observed on tomato fruits (Figure 1). Fruit symptoms began as small, dark, sunken lesions that had a water-soaked appearance, which increased in diameter and coalesced, leaving a larger sunken soft area. Lesions on ripe fruits became visible within one week of infection.

### 3.2. Fungal Isolation and Morphological Characterisation

**Figure 2:**
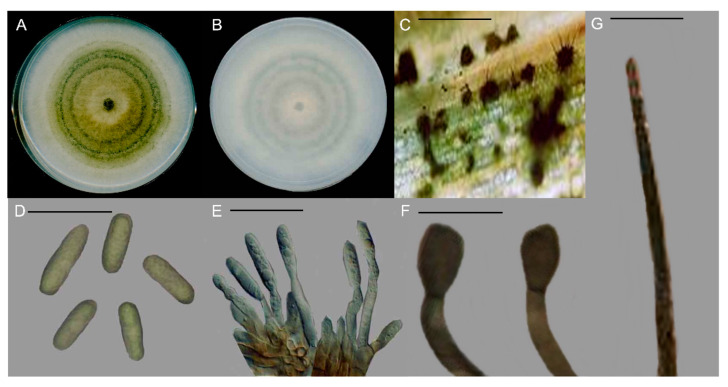
Morphological characteristics of *Colletotrichum truncatum*. (**A**) Colony appearance. (**B**) Pigmentation. (**C**) Acervuli. (**D**) Conidia. (**E**) Conidiogenous cell. (**F**) Appressoria. (**G**) Seta. Scale, (**C**,**E**,**G**) = 20 µm & (**D**,**F**) = 50 µm.

A total of 22 fungal isolates were recovered from tomato fruits showing anthracnose symptoms, and identified as *Colletotrichum* spp. through examination of macro- and microscopic characteristics. The colony was greenish-white, and pigmentation was greyish-white in color (Figure 2A,B). The average growth rate among the fungal isolates varied from 1.21 ± 0.27 to 1.67 ± 0.34 cm/d. Acervuli were scattered, irregularly shaped, and dark brown to black in color (Figure 2C). Conidia were hyaline, aseptate, and fusiform to rarely cylindrical, with the average size 13.4 to 18.9 × 5.2 to 7.3 µm (Figure 2D). Conidiogenous cells were hyaline, short, aseptate, and cylindrical, with sizes ranging from 11.2 to 16.33 × 4.6 to 5.7 µm (Figure 2E). Appressoria were simple, smooth, clavate to ovate, and dark brown, with sizes ranging from 10.2 to 14.6 × 7.6 to 9.4 µm (Figure 2F). Seta was dark brown, with tip more or less acute and acircular, ranging from 74.6 to 112.4 µm in size (Figure 2G).

### 3.3. Molecular Identification and Phylogenetic Analysis

Molecular identification based on the concatenated alignments of the ITS region, *tub2*, *gapdh*, *act,* and *cal* genes confirmed the identification of 22 fungal isolates collected from anthracnose symptomatic fruits of tomato. Based on the BLAST search, all the fungal isolates showed 99–100% sequence similarity to the isolates GQ485593 (ITS), GQ849429 (*tub2*), GQ856753 (*gapdh*), GQ856783 (*act*), and GQ849453 (*cal*) of *C. truncatum* (CBS 120709). The accession numbers of all the DNA sequences of the fungal isolates obtained in the present study are listed in Table 2.

**Figure 3:**
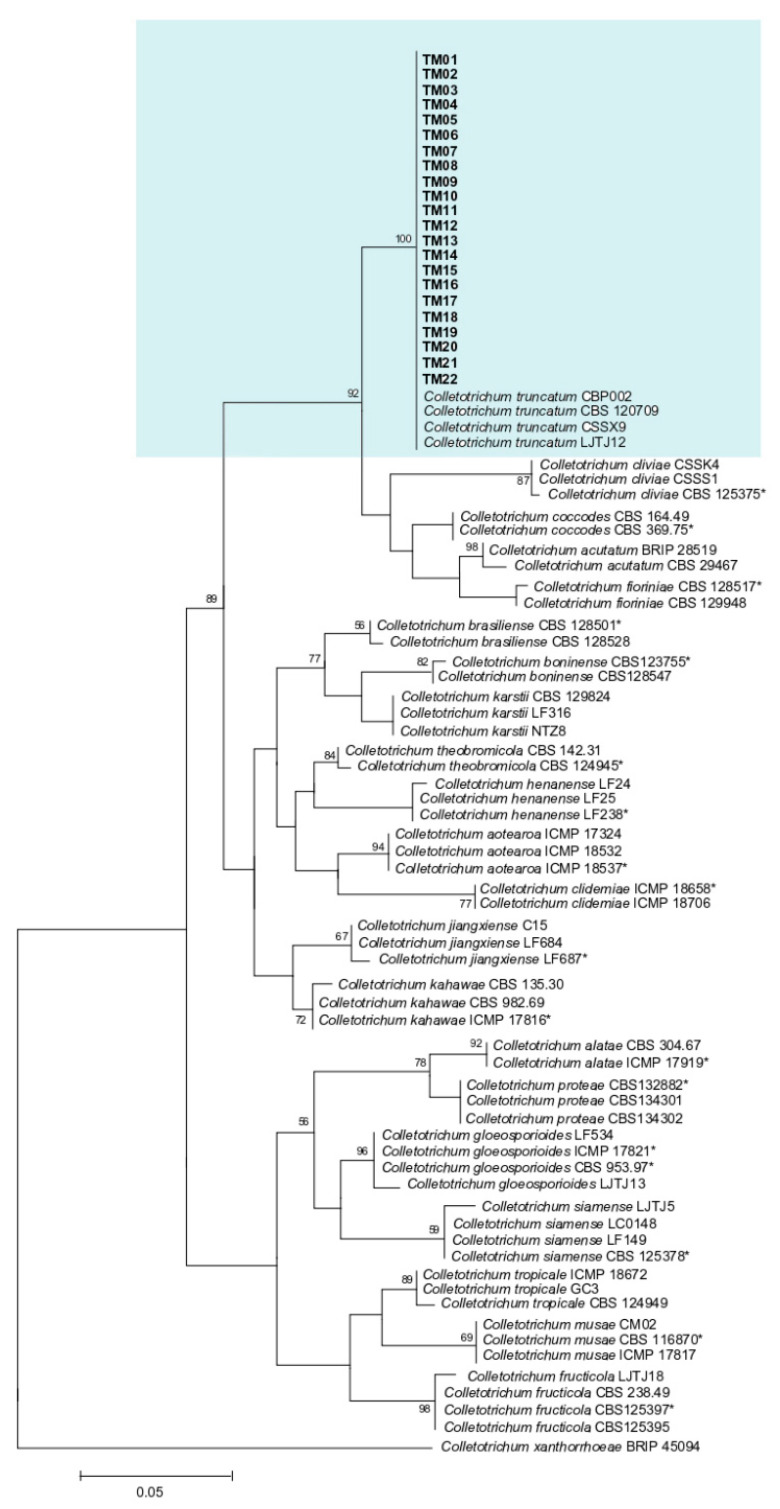
Maximum likelihood (ML) tree of *Colletotrichum truncatum* generated from analysis of the concatenated ITS region, *tub2*, *gapdh, act* and *cal* genes, with *Colletotrichum xantharrhoeae* as outgroup. Asterisks indicate ex-type isolates. The isolates used in the present study are indicated in bold font and highlighted in. Only bootstrap values > 50% are shown.

The phylogenetic tree derived from the combined ITS, *tub2*, *gapdh*, *act,* and *cal* sequences of *C. truncatum* showed that all 22 fungal isolates were clustered along with the reference strains of *C. truncatum* (CBP002, CBS 120709, CSSX9, and LJTJ12). The clade was supported by a bootstrap value of 100% (Figure 3).

### 3.4. Pathogenicity Assays

**Figure 4:**
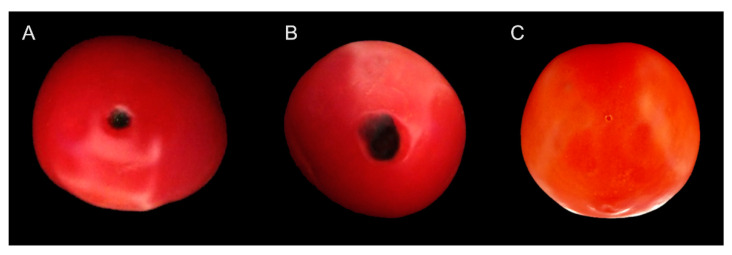
Pathogenicity of *Colletotrichum truncatum* on tomato fruits after 7 days of inoculation.

All the tested isolates of *Colletotrichum truncatum* were pathogenic on the tomato fruits by causing anthracnose lesions varying in size from 1.03 ± 0.13 to 1.46 ± 0.17 cm^2^ after 7 days of inoculation (Table 3). Symptoms of anthracnose and lesion sizes among the isolates of *C. truncatum* were significantly different (*p ˂ 0.05*). Initially, the inoculated tomato fruits showed small, circular to irregular dark chlorotic lesions, but After 7 days, the symptoms appeared as darker, sunken, and circular lesions, with the formation of concentric rings in the middle of the symptomatic areas which were similar to the field conditions (Figure 4A,B). The control experiments were asymptomatic (Figure 4C). The same fungal isolates were re-isolated from the symptomatic inoculated fruits of tomato, thus confirming *C. truncatum* as the pathogenic agent of anthracnose of tomato in Malaysia.

## 4. Discussion

A total of 22 fungal isolates associated with anthracnose of tomato fruits in the present study were identified as *Colletotrichum truncatum* through morphological and molecular characterisation. Although morphological characteristics are sufficient to distinguish between *Colletotrichum* species and fungi of other genera, inter-specific discrimination within the genus is often difficult as a result of overlaps in configuration of morphological features among identical *Colletotrichum* species [23,24,25]. This implies that the identification of *Colletotrichum* species only based on morphological distinctions may result in uncertainties in delineation of the genus [9,14].

A more precise approach will be the combination of morphological characteristics and molecular analysis for the accurate identification of *Colletotrichum* species [12]. A study of phylogenetic relationships could also reveal useful information on the genomic delineation of *C. truncatum*, which causes anthracnose of tomato. Thus, in the present study, multiple gene sequence alignments of ITS, *tub2*, *gapdh*, *act* and *cal* were shown to be effective in identifying *C. truncatum* from anthracnose of tomato. In related studies, Liu et al. [13] and Weir et al. [14] also used those five conserved genes to accurately identify and resolve the phylogenetic status of *Colletotrichum* species.

The present study highlighted the occurrence of tomato anthracnose in Malaysia. All the isolates of *C. truncatum* isolated in the present study caused anthracnose of tomato with varying degrees of severity. Although *C. boninense* was earlier reported to be associated with tomato anthracnose in Pahang, Malaysia [26], this study is the first report of tomato anthracnose caused by *C. truncatum* in Malaysia. Other reports of tomato anthracnose caused by *C. truncatum* have been published in China [27], India [28] and Trinidad [29].

Generally, *Colletotrichum* is a genus of diverse plant pathogenic fungi which causes diseases in a wide variety of plant species worldwide, and several *Colletotrichum* species have the capacity to infect a single host-plant, and a single *Colletotrichum* species is also capable of infecting several hosts [2,3,4,5]. A broad range of host species including avocado, chilli, mango, olive, papaya, strawberry, and watermelon, can be infected by different *Colletotrichum* species worldwide [30,31,32,33,34,35,36]. Anthracnoses caused by *Colletotrichum* spp. are important diseases in Malaysia, infecting numerous hosts such as banana, chilli, dragon fruit, eggplant, and watermelon [37,38,39,40,41]. Previous reports also identified *Colletotrichum acutatum*, *C. coccodes*, *C. dematium*, and *C. gloeosporioides* as the causative agents of tomato anthracnose globally [16,42].

## 5. Conclusions

In the present study, morphological traits coupled with multigene phylogenetic analysis were effective in identifying *C. truncatum* as the fungal species associated with diseased tomato fruits showing symptoms of anthracnose in Malaysia. Pathogenicity tests further revealed that *C. truncatum* was the causative agent of anthracnose of tomato fruits. This confirms that *C. truncatum* is an emerging pathogen that is capable of causing anthracnose disease which may threaten the yield and profitability of tomato production as well as the other crops in regions where it has already been established. Information on disease symptomatology, etiology, epidemiology and pathogenesis provided by this study could be useful in disease monitoring and formulation of strategies for effective management of anthracnose, thus reducing yield losses of tomatoes.

## Figures and Tables

**Table 1:** Primers used for PCR amplifications and DNA sequencing.

Gene	Primer	Sequence (5′–3′)	Reference
ITS	ITS1 ITS4	TCCGTAGGTGAACCTGCGG TCCTCCGCTTATTGATATGC	[17]
*tub2*	Bt2a Bt2b	GGTAACCAAATCGGTGCTGCTTTC ACCCTCAGTGTAGTGACCCTTGGC	[18,19]
*gapdh*	GDF1 GDR1	GCCGTCAACGACCCCTTCATTGA GGGTGGAGTCGTACTTGAGCATGT	[20]
*act*	ACT-512F ACT-783R	ATGTGCAAGGCCGGTTTCGC TACGAGTCCTTCTGGCCCAT	[21]
*cal*	CAL-228F CAL-737R	GAGTTCAAGGAGGCCTTCTCCC CATCTTTCTGGCCATCATGG	[21]

**Table 2:** Fungal isolates obtained from the present study and reference species used for sequence comparisons and phylogenetic analysis of *Colletotrichum truncatum*.

Species	Isolate	Host	Location	GenBank Accession Number				
				ITS	*tub2*	*gapdh*	*act*	*cal*
*Colletotrichum truncatum*	TM01	*Solanum lycopersicum*	Malaysia	OP456600	OP495634	OP495656	OP495678	OP495700
	TM02	*Solanum lycopersicum*	Malaysia	OP456601	OP495635	OP495657	OP495679	OP495701
	TM03	*Solanum lycopersicum*	Malaysia	OP456602	OP495636	OP495658	OP495680	OP495702
	TM04	*Solanum lycopersicum*	Malaysia	OP456603	OP495637	OP495659	OP495681	OP495703
	TM05	*Solanum lycopersicum*	Malaysia	OP456604	OP495638	OP495660	OP495682	OP495704
	TM06	*Solanum lycopersicum*	Malaysia	OP456605	OP495639	OP495661	OP495683	OP495705
	TM07	*Solanum lycopersicum*	Malaysia	OP456606	OP495640	OP495662	OP495684	OP495706
	TM08	*Solanum lycopersicum*	Malaysia	OP456607	OP495641	OP495663	OP495685	OP495707
	TM09	*Solanum lycopersicum*	Malaysia	OP456608	OP495642	OP495664	OP495686	OP495708
	TM10	*Solanum lycopersicum*	Malaysia	OP456609	OP495643	OP495665	OP495687	OP495709
	TM11	*Solanum lycopersicum*	Malaysia	OP456610	OP495644	OP495666	OP495688	OP495710
	TM12	*Solanum lycopersicum*	Malaysia	OP456611	OP495645	OP495667	OP495689	OP495711
	TM13	*Solanum lycopersicum*	Malaysia	OP456612	OP495646	OP495668	OP495690	OP495712
	TM14	*Solanum lycopersicum*	Malaysia	OP456613	OP495647	OP495669	OP495691	OP495713
	TM15	*Solanum lycopersicum*	Malaysia	OP456614	OP495648	OP495670	OP495692	OP495714
	TM16	*Solanum lycopersicum*	Malaysia	OP456615	OP495649	OP495671	OP495693	OP495715
	TM17	*Solanum lycopersicum*	Malaysia	OP456616	OP495650	OP495672	OP495694	OP495716
	TM18	*Solanum lycopersicum*	Malaysia	OP456617	OP495651	OP495673	OP495695	OP495717
	TM19	*Solanum lycopersicum*	Malaysia	OP456618	OP495652	OP495674	OP495696	OP495718
	TM20	*Solanum lycopersicum*	Malaysia	OP456619	OP495653	OP495675	OP495697	OP495719
	TM21	*Solanum lycopersicum*	Malaysia	OP456620	OP495654	OP495676	OP495698	OP495720
	TM22	*Solanum lycopersicum*	Malaysia	OP456621	OP495655	OP495677	OP495699	OP495721
	CBP002	*Brassica parachinensis*	China	KF030677	KF240819	KF300886	KF158412	KF114851
	CBS 120709	*Capsicum frutescens*	India	GQ485593	GQ849429	GQ856753	GQ856783	GQ849453
	CSSX9	*Hymenocallis americana*	China	GQ485594	GQ849436	GQ856752	GQ856772	GQ849461
	LJTJ12	*Capsicum* sp.	China	KP748203	KP823843	KP823782	KP823765	KP823834
*Colletotrichum acutatum*	BRIP 28519	*Carica papaya*	Australia	FJ972601	FJ907443	FJ972580	FJ907428	FJ917510
	CBS 29467	*Carica papaya*	Australia	FJ972610	FJ907444	FJ972581	FJ907429	FJ917511
*Colletotrichum alatae*	CBS 304.67	*Dioscorea alata*	Nigeria	JX010191	JX010449	JX010011	JX009470	JX009739
	ICMP 17919 *	*Dioscorea alata*	India	JX010190	JX010383	JX009990	JX009471	JX009738
*Colletotrichum aotearoa*	ICMP 17324	*Kunzea ericoides*	New Zealand	JX010198	JX010418	JX009991	JX009538	JX009619
	ICMP 18532	*Vitex lucens*	New Zealand	JX010220	JX010421	JX009906	JX009544	JX009614
	ICMP 18537 *	*Coprosma* sp.	New Zealand	JX010205	JX010420	JX010005	JX009564	JX009611
*Colletotrichum boninense*	CBS 123755 *	*Crinum asiaticum*	Japan	JQ005153	JQ005588	JQ005240	JQ005501	JQ005674
	CBS 128547	*Camellia* sp.	New Zealand	JQ005159	JQ005593	JQ005249	JQ005507	JQ005680
*Colletotrichum brasiliense*	CBS 128501 *	*Passiflora edulis*	Brazil	JQ005235	JQ005669	JQ005322	JQ005583	JQ005756
	CBS 128528	*Passiflora edulis*	Brazil	JQ005234	JQ005668	JQ005321	JQ005582	JQ005755
*Colletotrichum clidemiae*	ICMP 18658 *	*Clidemia hirta*	USA	JX010265	JX010438	JX009989	JX009537	JX009645
	ICMP 18706	*Vitis* sp.	USA	JX010274	JX010439	JX009909	JX009476	JX009639
*Colletotrichum cliviae*	CBS 125375 *	*Clivia miniate*	China	JX519223	JX519249	JX546611	JX519240	KX957765
	CSSK4	*Clivia miniate*	China	GQ485607	GQ849440	GQ856756	GQ856777	GQ849464
	CSSS1	*Clivia miniate*	China	GU109479	GU085869	GU085868	GU085861	GU085864
*Colletotrichum coccodes*	CBS 164.49	*Solanum tuberosum*	Netherlands	HM171678	KU821197	HM171672	HM171666	HM171669
	CBS 369.75 *	*Solanum tuberosum*	Netherlands	HM171679	KU821198	HM171673	HM171667	HM171670
*Colletotrichum fioriniae*	CBS 128517 *	*Fiorinia externa*	USA	JQ948292	JQ949943	JQ948622	JQ949613	MN895526
	CBS 129948	*Tulipa* sp.	UK	JQ948344	JQ949995	JQ948674	JQ949665	MN895531
*Colletotrichum fructicola*	CBS 238.49	*Ficus habrophylla*	Germany	JX010181	JX010400	JX009923	JX009495	JX009671
	CBS 125395	*Theobroma cacao*	Panama	JX010172	JX010408	JX009992	JX009543	JX009666
	CBS 125397 *	*Tetragastris panamensis*	Panama	JX010173	JX010409	JX010032	JX009581	JX009674
	LJTJ18	*Capsicum* sp.	China	KP748209	KP823856	KP823788	KP823744	KP823814
*Colletotrichum gloeosporioides*	CBS 953.97 *	*Citrus sinensis*	Italy	GQ485605	GQ849434	GQ856762	GQ856782	GQ849452
	ICMP 17821 *	*Citrus sinensis*	Italy	JX010152	JX010445	JX010056	JX009531	JX009731
	LF534	*Camellia sinensis*	China	KJ955158	KJ955305	KJ954859	KJ954434	KJ954710
	LJTJ13	*Capsicum* sp.	China	KP748204	KP823863	KP823783	KP823751	KP823821
*Colletotrichum henanense*	LF24	*Cirsium japonicum*	China	KM610182	KM610184	KM610178	KM610172	KM610176
	LF25	*Cirsium japonicum*	China	KM610183	KM610185	KM610179	KM610173	KM610177
	LF238 *	*Camellia sinensis*	China	KJ955109	KJ955257	KJ954810	KM023257	KJ954662
*Colletotrichum jiangxiense*	C15	*Citrus sinensis*	China	MT318946	MT602355	MT602358	MT602346	KJ954701
	LF684	*Camellia sinensis*	China	KJ955198	KJ955345	KJ954899	KJ954469	KJ954749
	LF687 *	*Camellia sinensis*	China	KJ955201	KJ955348	KJ954902	KJ954471	KJ954752
*Colletotrichum kahawae*	CBS 135.30	*Coffea* sp.	Kenya	JX010235	JX010432	JX010037	JX009554	JX009640
	CBS 982.69	*Coffea arabica*	Angola	JX010234	JX010435	JX010040	JX009474	JX009638
	ICMP 17816 *	*Coffea arabica*	Kenya	JX010231	JX010444	JX010012	JX009452	JX009642
*Colletotrichum karstii*	CBS 129824	*Musa* sp.	Colombia	JQ005215	JQ005649	JQ005302	JQ005563	JQ005736
	LF316	*Camellia sinensis*	China	KJ955125	KJ955273	KJ954826	KJ954405	KY971492
	NTZ8	*Nandina domestica*	China	MH152578	MH152594	MH152586	MH152582	MH152598
*Colletotrichum musae*	CBS 116870 *	*Musa* sp.	USA	JX010146	HQ596280	JX010050	JX009433	JX009742
	CM02	*Musa* x *paradisiaca*	Brazil	MH746945	MH746949	MH746948	MH622522	MH746946
	ICMP 17817	*Musa sapientum*	Kenya	JX010142	JX010395	JX010015	JX009432	JX009689
*Colletotrichum proteae*	CBS 132882 *	*Protea* sp.	South Africa	KC297079	KC297101	KC297009	KC296940	KC296960
	CBS 134301	*Protea* sp.	South Africa	KC842385	KC842387	KC842373	KC842373	KC842375
	CBS 134302	*Protea* sp.	South Africa	KC842386	KC842388	KC842380	KC842374	KC842376
*Colletotrichum siamense*	CBS 125378 *	*Hymenocallis americana*	China	JX010278	JX010410	JX010019	JX009441	JX009709
	LC0148	*Camellia* sp.	China	KJ955078	KJ955227	KJ954779	KJ954360	KJ954631
	LF149	*Camellia* sp.	China	KJ955089	KJ955238	KJ954790	KJ954371	KJ954642
	LJTJ5	*Capsicum* sp.	China	KP748195	KP823868	KP823756	KP823775	KP823825
*Colletotrichum theobromicola*	CBS 142.31	*Fragaria* x *ananassa*	USA	JX010286	JX010373	JX010024	JX009516	JX009592
	CBS 124945 *	*Theobroma cacao*	Panama	JX010294	JX010447	JX010006	JX009444	JX009591
*Colletotrichum tropicale*	CBS 124949	*Theobroma cacao*	Panama	JX010264	JX010407	JX010007	JX009489	JX009719
	GC3	*Vitis* sp.	Taiwan	MT555315	MT648526	MT648519	MT648522	MT062402
	ICMP 18672	*Litchi chinensis*	Japan	JX010275	JX010396	JX010020	JX009480	JX009722
*Colletotrichum xanthorrhoeae*	BRIP 45094	*Xanthorrhoea* sp.	Australia	JX010261	JX010448	JX009927	JX009478	JX009653

* ex-type isolate.

**Table 3:** Lesion areas produced by *C. truncatum* isolates on inoculated fruits of tomato.

Fungal Species	Isolate Code	* Lesion Area (cm^2^)
*C. truncatum*	TM01	1.12 ± 0.17 ^ab^
	TM02	1.09 ± 0.07 ^ab^
	TM03	1.25 ± 0.12 ^a^
	TM04	1.21 ± 0.19 ^a^
	TM05	1.07 ± 0.05 ^ab^
	TM06	1.46 ± 0.17 ^a^
	TM07	1.19 ± 0.11 ^a^
	TM08	1.36 ± 0.21 ^a^
	TM09	1.28 ± 0.07 ^a^
	TM10	1.34 ± 0.11 ^a^
	TM11	1.03 ± 0.13 ^ab^
	TM12	1.15 ± 0.19 ^a^
	TM13	1.27 ± 0.14 ^a^
	TM14	1.04 ± 0.13 ^ab^
	TM15	1.23 ± 0.21 ^a^
	TM16	1.41 ± 0.09 ^a^
	TM17	1.16 ± 0.11 ^a^
	TM18	1.34 ± 0.19 ^a^
	TM19	1.10 ± 0.05 ^ab^
	TM20	1.19 ± 0.13 ^a^
	TM21	1.26 ± 0.05 ^a^
	TM22	1.07 ± 0.10 ^ab^
Control		0 ± 0 ^b^

* Means ± standard deviations followed by different letters within the column are significantly different (*p* ˂ 0.05).

## Data Availability

Not applicable.
